# The Lighting of Hospitals

**Published:** 1911-02-04

**Authors:** 


### The Lighting of Hospitals.

### GAS AND ELECTRIC PLANT.

The lighting of hospitals is perhaps one of the most important subjects that a committee or a superintendent have to consider. The problem naturally divides itself into two branches—namely, the lighting of large hospitals and the lighting of small ones, such as cottage hospitals. Large hospitals are nearly always situated in, or in the neighbourhood of, large towns, where both gas and electricity are laid on, and the question of lighting then resolves itself into which of these two shall be employed. Cottage hospitals and sanatoria, on the other hand, are often situated in out-of-the-way places where there is no supply of either gas or electricity, and for them other sources of light have to be provided.

### I.—The Lighting of Large Hospitals.

In the case of large hospitals, if lighting by gas is decided upon, the best method, both for convenience and economy, is usually to take a supply from the town service. If the electric light is chosen the current may also be taken from the town electric supply service, or if it be preferred it may be generated within the hospital premises. Usually it will be found more economical and more convenient to take a supply from the local service. In very few cases have town hospitals laid down their own electric lighting plant, and where it has been done the cost of the current has been considerably greater than it otherwise need have been.

During the last 28 years gas has been improving at a very rapid rate. At the Electrical Exhibition of 1882 gas was very bad and very dear, and one of the principal reasons for the development of the electric light at that date was the hope that it would furnish a better light at a cheaper rate than gas. Since then the improvement in gas has been continuous, and the cost has been steadily decreasing.

### The Welsbach.

The invention of the Welsbach mantle some twenty years back, after repeated failures, enabled gas to compete upon equal, and latterly upon more than equal, terms with electricity. On the other hand, many difficulties that were not foreseen have developed in the generation of electricity, increasing its cost above that which was estimated. During the last few years electricity has again taken a step in advance by the invention of the metallic filament lamp; but, also during the same period, gas engineers have developed the inverted mantle, and, for large lights, the high-pressure system. The electric light has the enormous advantage that it is perfectly steady, while the Welsbach mantle, even in its latest form, flickers very much. To anyone who has to work by artificial light for any length of time the difference between the two will be painfully apparent.

The Welsbach inverted mantle is now made to give from 20 c.p. upwards, but the form commonly employed gives 60 c.p. for a consumption of 4 cubic feet per hour. The metallic filament electric incandescent lamp is now also made for 16 c.p. and upwards, and for any pressure and for any form of current. It furnishes its light for a consumption of 1¼ watt per candle, or rather less than one-third of that taken by the old carbon lamps.

### The Cost.

The cost of the lighting of a hospital of any size depends upon the number of lights that are required and the cost at which the current or the gas is supplied. The cost of the individual light per 1,000 hours may be found by the following simple rule, whether it be electricity or gas. Multiply the number of cubic feet per hour consumed in the case of gas, or the number of watts in the case of electricity, by the price of the gas per 1,000 cubic feet, or by that of current per Board of Trade unit, and add the cost of mantles or lamps used up during the 1,000 hours. Taking the case of the 60 c.p. inverted Welsbach mantle, consuming 4 ft. of gas per hour, and taking the cost at 2s. 6d. per 1,000 feet, and the cost of mantles at 3d. each, the cost of each light for each 1,000 hours works out at 10s. 6d. Gas engineers claim 60 c.p. for a consumption of 2 cubic feet per hour, with the latest forms of inverted mantles. This would reduce the cost to 5s. 6d. per 1,000 hours.

### Some Figures Compared.

In the case of the 50 c.p. metallic filament incandescent lamp, which would be approximately the equivalent of the 60 c.p. inverted Welsbach mantle, and taking the cost of current at 3d. per Board of Trade unit, and the cost of renewing the lamps at 3s. 6d. each, the total cost of the individual lamp for 1,000 hours works out to 22s. 7½d. If 25 c.p. metallic filament lamps could be employed, the cost would be about half that, or about the same as the cost of the 60 c.p. Welsbach incandescent mantle, taking 4 cubic feet per hour.

### The Cost per Annum.

It will be a question for individual hospital superintendents and governing bodies as to whether 50 c.p. metallic filament lamps shall be employed, or 25, or how many. The general tendency in the matter of illumination is to give more light, to make the conditions during which artificial light is employed more and more nearly approach those of daylight.

The total cost of lighting any given hospital per annum may be found by multiplying the number of lights in the hospital by the average number of hours of burning, and by the figures found by the above method. The number of hours of burning will vary with different hospitals. The cost of fitting gas in hospital buildings may be taken at approximately 10s. per burner, and that of the electric light about 20s. per lamp.

